# Root exudation and root development of lettuce (*Lactuca sativa* L. cv. Tizian) as affected by different soils

**DOI:** 10.3389/fmicb.2014.00002

**Published:** 2014-01-24

**Authors:** G. Neumann, S. Bott, M. A. Ohler, H.-P. Mock, R. Lippmann, R. Grosch, K. Smalla

**Affiliations:** ^1^Department of Nutritional Crop Physiology, Institute of Crop Science (340h), University of HohenheimStuttgart, Germany; ^2^Leibniz-Institut für Pflanzengenetik und KulturpflanzenforschungGatersleben, Germany; ^3^Department Plant Health, Leibniz Institute of Vegetable and Ornamental Crops Großbeeren/Erfurt e.V.Großbeeren, Germany; ^4^Julius Kühn-Institut Federal Research Centre for Cultivated Plants, Institute for Epidemiology and Pathogen DiagnosticsBraunschweig, Germany

**Keywords:** lettuce, root exudates, root morphology, soil effects

## Abstract

Development and activity of plant roots exhibit high adaptive variability. Although it is well-documented, that physicochemical soil properties can strongly influence root morphology and root exudation, particularly under field conditions, a comparative assessment is complicated by the impact of additional factors, such as climate and cropping history. To overcome these limitations, in this study, field soils originating from an unique experimental plot system with three different soil types, which were stored at the same field site for 10 years and exposed to the same agricultural management practice, were used for an investigation on effects of soil type on root development and root exudation. Lettuce (*Lactuca sativa* L. cv. Tizian) was grown as a model plant under controlled environmental conditions in a minirhizotrone system equipped with root observation windows (rhizoboxes). Root exudates were collected by placing sorption filters onto the root surface followed by subsequent extraction and GC-MS profiling of the trapped compounds. Surprisingly, even in absence of external stress factors with known impact on root exudation, such as pH extremes, water and nutrient limitations/toxicities or soil structure effects (use of sieved soils), root growth characteristics (root length, fine root development) as well as profiles of root exudates were strongly influenced by the soil type used for plant cultivation. The results coincided well with differences in rhizosphere bacterial communities, detected in field-grown lettuce plants cultivated on the same soils (Schreiter et al., this issue). The findings suggest that the observed differences may be the result of plant interactions with the soil-specific microbiomes.

## Introduction

Root exudates of higher plants with nutritional, signaling, and antibiotic functions are shaping rhizosphere-microbial communities, which in turn can exert stimulatory or inhibitory effects on plant growth and development. Already Hiltner (1904) postulated that specific patterns of root exudation in different plant species may recruit a specific rhizosphere microflora, which may comprise beneficial partners but also pathogens as uninvited guests. He also pointed out that a more detailed knowledge of these interactions may open perspectives for practical applications in agriculture and plant protection. Nowadays, the availability of novel techniques for the characterization of microbial communities by high throughput sequencing approaches, metabolomics and the development of non-destructive, localized exudate sampling techniques (Neumann et al., 2009; Bakker et al., 2012; Chaparro et al., 2013) opens the way for a more detailed and comprehensive look on the interactions between rhizosphere microbiomes and roots of their host plants. During the last two decades enormous progress was achieved in the characterization of factors determining root exudation, which exhibits high variability within different plant species and even cultivars, within different root zones and developmental stages of individual plants and in response to various biotic and abiotic stress factors (Neumann, 2007; Neumann and Römheld, 2007; Badri and Vivanco, 2009). An increasing number of investigations meanwhile also address the interactions between root exudates and corresponding changes of the microflora in the rhizosphere (Marschner et al., 2002; Weisskopf et al., 2006; Bakker et al., 2012).

In the present study, we hypothesized that different soil types with different physicochemical properties will influence root growth patterns and root exudation, which in turn may have an impact on composition and function of rhizosphere-microbial communities (see Schreiter et al., this issue). The enormous plasticity of root growth and root exudation in response to different soil conditions and particularly to stress factors, such as nutrient limitation, mineral toxicities, and extremes in soil moisture and soil structure, is a well-described phenomenon (Neumann and Römheld, 2002, 2007; Neumann, 2007). In our study, we used field soils originating from a unique experimental plot system with three different soil types, which were stored at the same field site for 10 years under the same agricultural management and used in parallel by Schreiter et al. (this issue) to characterize the influence of soil type on rhizosphere-bacterial communities under field conditions. This approach offered the opportunity to study the influence of soil properties independent from cropping history or climatic factors.

Lettuce plants (cv. Tizian) were grown in minirhizotrons, equipped with root observation windows (Neumann, 2006a), which allowed root growth monitoring and localized collection of root exudates and rhizosphere soil solution from defined root zones by use of sorption filters (Neumann, 2006b; Haase et al., 2007) with subsequent re-extraction and GC-MS profiling of the exudate patterns (Lippmann et al., 2009). Due to the limited available field plot size, unfortunately direct collection of root exudates under field conditions by use of root windows (Neumann et al., 2009) was not possible.

## Materials and methods

### Plant cultivation

**Figure 1:**
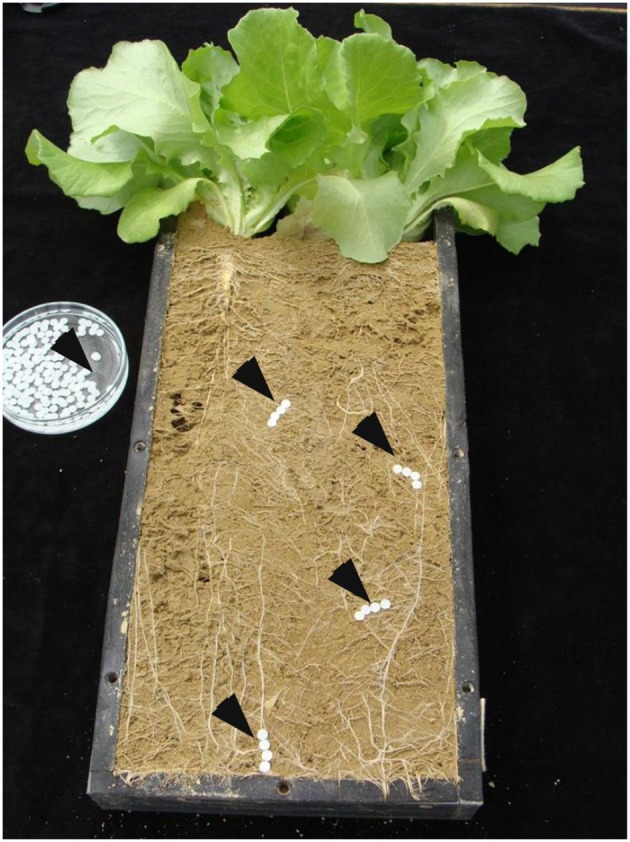
**Lettuce plants *Lactuca sativa* L. cv. Tizian (BBCH 19) grown on loess loam**. Root observation window of a minirhizotron (rhizobox) prepared for exudate collection with sorption filters (indicated by black arrows).

*Lactuca sativa* L. cv. Tizian seedlings were pre-cultivated until the 2-leaf stage (BBCH 12) in peat culture substrate sand mixture (7:3 w/w; TKS1-Anzuchtsubstrat, Floragard, Germany). Thereafter, the seedlings were transferred to minirhizotrons (rhizoboxes) with transparent root observation windows (36 × 11.5 × 2.5 cm; Neumann, 2006a) filled each with 1 kg of the selected test soils (Figure 1).

The experimental system included three soils of different origin: Arenic-Luvisol with less silty sand and 5.5% clay [diluvial sand (DS)], Gleyic-Fluvisol with heavy sandy loam and 27.5% clay [alluvial loam (AL)], and Luvic-Phaeozem with medium content of clayey silt and 17.2% clay [loess loam (LL)]. Soil properties are summarized in Table 1.

**Table 1:** **Characteristics of the field soils used for the experiments**.

**Soil type**	**pH**	**Ct [%]**	**TRD [g/cm^3^]**	**FAT [%]**	**P CAL. [mg/100 g]**	**K CAL. [mg/100 g]**
Loess loam	7.3	1.90	1.36	23.6	26.1	30.0
Diluvial sand	6.1	0.80	1.46	7.7	25.4	12.3
Alluvial loam	6.7	1.80	1.31	34.9	46.0	26.0

Ct, total carbon content; TRD, bulk density; FAT, fine grained particles/ particles <6.3 μm.

To exclude nutrient limitation, prior to transplanting, the soils received a basal macronutrient fertilization of N, P, K, and Mg. Nitrogen was applied as CaNO_3_ at 100 mg N kg^−1^ soil, P as Ca(H_2_PO_4_)_2_ at 100 mg P kg^−1^ soil, K as K_2_SO_4_ at 150 mg K kg^−1^ soil and Mg as MgSO_4_ at 50 mg Mg kg^−1^ soil. Final soil moisture level was adjusted to 18–20% w/w and controlled gravimetrically during the culture period.

Rhizoboxes were fixed at an angle of 45° to promote root development along the observation windows and plants were cultivated until BBCH 19 in a growth chamber with a 16 h light period (200 μmol m^−2^ s^−1^), 60% rel. humidity and a day/night temperature of 25°/23° C.

### Final harvest and exudate sampling

Prior to final harvest (BBCH 19; 5 weeks after transplanting), the root observation windows were opened and root exudates were collected by placing sorption filters onto the surface of 2 cm sub-apical root zones (Figure 1) according to the method described by Haase et al. (2007). The time point for exudate sampling was selected since root growth, shoot to root carbohydrate partition and root exudation usually is particularly intense during vegetative growth and declines after entering the generative phase (Marschner, 1995). The collection period was 4 h and the filters were subsequently stored at −20°C until further analysis. Thereafter, roots were washed out of the soil using sieves with 1 and 0.5 mm mesh size. Roots were dried on filter paper and fresh biomass was recorded. Thereafter, the root samples were stored in 30% (v/v) ethanol until further analysis. Biomass of the shoot material was recorded and dried at 60° C for mineral nutrient analysis. Root analysis was performed using the WinRhizo system (Regent Instruments, Quebec, Canada).

### Mineral nutrient analysis

For determination of the plant-nutritional status, 500 mg of dried leaf material was ashed in a muffle furnace at 500°C for 5 h. After cooling, the samples were extracted twice with 2 mL of 3.4 M HNO_3_ until dryness to precipitate SiO_2_. The ash was dissolved in 2 mL of 4 M HCl, subsequently diluted ten times with hot deionized water, and boiled for 2 min. After addition of 0.1 mL Cs/La buffer to 4.9 mL ash solution, Fe, Mn, and Zn concentrations were measured by atomic absorption spectrometry (UNICAM 939, Offenbach/Main, Germany).

### Exudate profiling

The extraction of the sorption filters containing root exudates and rhizosphere soil solution was conducted with 80% methanol. Filters were removed by centrifugation, and the supernatant was evaporated to dryness at 30°C using a speed vac concentrator (Savant, Farmington, USA) and stored at −80°C until further analysis. For analysis, supernatants were re-dissolved in 200 μL methanol, transferred into GC-MS glass vials and evaporated to dryness at 30°C. Derivatisation was performed online directly prior to injection using a MPS Autosampler (Gerstel, Mühlheim a. d. R., Germany) by adding 25 uL methoxyhydroxymethylamine (20 mg mL^−1^ in pyridine) and incubation for 2 h at 37°C, 350 rpm. Thereafter, 50 μL MSTFA including standard alcanes from Sigma C7–C30 (0.1% v/v) were added and incubated for 30 min at 37°C, 350 rpm.

One μL aliquots were analyzed by an Agilent 7890 gas chromatograph (Agilent, Santa Clara, CA, USA) in the splitless mode, coupled to a TOF mass spectrometer GCT Premier (Waters Corporation, Eschborn, Germany). Separation was performed on a RxiⓇ5Sil MS Integra column (Restek) with 0.25 mm inner diameter and 0.25 μm film thickness, including a 5 m guard column according to Lippmann et al. (2009). Injection temperature was 240°C. The temperature program for GC separation was: 3 min 80°C isothermal followed by a ramp of 5°C min^−1^ to 300°C for 5 min. Mass spectroscopical (MS) data were recorded with Mass Lynx 4.1 (Waters Corporation) at a rate of 10 spectra s^−1^ in a range of 50–700 m/z. Metabolites were identified automatically with the internal software ChromaLynx using the NIST 5 library and interesting components were verified manually by comparison with reference spectra. For principal component analysis (PCA) MarkerLynx (Waters Corporation) was used with following settings: 20 masses per retention time in the range of 100–330 m/z and a tolerance of 0.05 Da were isolated at a threshold at 5% of base peak intensity. Pareto algorithm was used for visualization.

## Results

### Root growth characteristics

Biomass production and particularly root length were significantly influenced by the selected soil types (Table 2). Root growth characteristics of lettuce plants grown on DS and LL were significantly different from AL. Total root length on AL, mainly represented by fine roots (diameter < 0.4 mm, 70%) was 2.5–4 fold increased as compared with DS and LL, respectively. This was also reflected in a significantly higher (fine) root length, root biomass and a lower average root diameter of lettuce plants grown on AL. However, differences in root growth were not associated with corresponding differences in shoot biomass production and surprisingly, the highest shoot biomass was detected in lettuce plants grown on LL with development of the smallest root system.

**Table 2:** **Biomass production and root characteristics of *Lactuca sativa* L. cv. Tizian (BBCH19), grown on three different soils**.

**Soil type**	**Shoot fresh weight [g plant^−1^]**	**Root fresh weight [g plant^−1^]**	**Total root length [cm plant^−1^]**	**Fine root length [cm plant^−1^]0–0.4mmdiameter**	**Average root diameter [mm]**
Alluvial loam	6.67 ± 1.07a	0.84 ± 0.18a	562.2 ± 92.2a	391.0 ± 58.3a	0.35 ± 0.01a
Loess loam	9.07 ± 1.02a	0.30 ± 0.04b	126.8 ± 14.9b	87.3 ± 12.5b	0.49 ± 0.02b
Diluvial sand	5.32 ± 0.60a	0.24 ± 0.05b	197.1 ± 27.5b	150.9 ± 18.2b	0.43 ± 0.01b

Means ± SE of four independent replicates. In each column different characters indicate significant differences (One-way ANOVA, Holm-Sidak test, p = 0.05).

### Root exudates

At BBCH 19 (5 weeks after transplanting), root development along the root observation window was sufficiently expressed to enable the collection of root exudates and rhizosphere soil solution from defined sub-apical root zones in young actively growing roots (Figure 1), which have been identified in many earlier studies as the sites of the most intense expression of root exudation, nutrient uptake and root-induced rhizosphere-chemical changes (for review see Neumann and Römheld, 2007).

In total 33 compounds were identified by GC-MS profiling in the soil solutions collected with sorption filters from the root surface and rhizosphere of 2 cm sub-apical root zones, comprising 17 amino acids and amides, 8 sugars, and sugar alcohols, 5 organic acids as well as ornithine, urea, and phosphate (Table 3). Benzoic and lauric acids were detected as exudate compounds with allelopathic and antibiotic properties (Walters et al., 2003; Lee et al., 2006; Yoon et al., 2012).

**Table 3:** **List of low molecular-weight compounds detected by GC-MS root exudate profiling from roots of *Lactuca sativa* cv Tizian (BBCH19) grown on three different soils**.

**Chemical group**	**Compound**	**Loess loam**	**Alluvial loam**	**Diluvial sand**
Amino acids and amines	Alanine	+	+	++
	beta-Alanine	+	−	+
	Aspartate	+	+	+
	Glutamate	−	−	+
	Glutamine	+	+	+
	Glycine	+++	++	++
	Leucine	++	+	+
	Isoleucine	+	+	+
	Proline	+	+	+
	4-Hydroxyproline	+	+	+
	Pyroglutamate	++	+	+
	Serine	++	++	+
	Threonine	++	+	+
	Valine	++	+	++
	beta-Aminobutyric acid	+	+	+
	4-Aminobutyric acid	+	−	+
	Putrescine	+	+	+
Sugars and sugar alcohols	Glucose	+++	+	++
	Fructose	+++	+	++
	Mannose	+	−	−
	Maltose	+++	+	+++
	Trehalose	+++	+	+++
	Sucrose	+++	++	++
	Glycerol	+++	+++	+++
	Inositol	+++	+	+
Organic acids	Malate	+	+	+
	Fumarate	+	+	+
	Succinate	++	+++	++
	Lauric acid	++	+	+
	Benzoic acid	++	++	++
Others	Urea	+++	++	++
	Phosphate	+	+	+
	Ornithine	+	+	+

(−, absent; +, low; ++, medium; +++, high).

**Figure 2:**
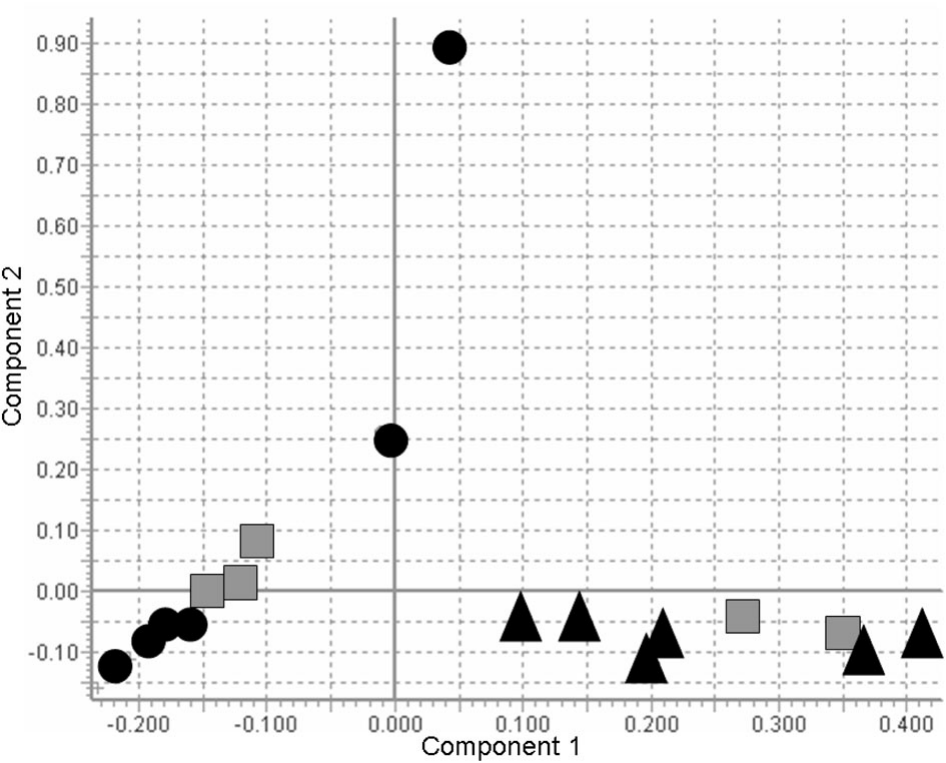
**Principal component analysis (PCA) of the GC-MS root exudate profiles collected from 2 cm-subapical root zones of *Lactuca sativa* L. cv. Tizian BBCH19 grown on three different soils (Loess loam** 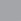; **Alluvial loam ▲; Diluvial sand ●)**.

PCA analysis revealed clear differences in the root exudate profiles collected from lettuce plants grown on the different soils (Figure 2), with DS separated from AL. However, with the exception of several amino acids (beta-alanine, glutamate, 4-amino butyric acid), the differences were rather quantitative than qualitative (Table 3).

Interesting quantitative differences were observed for low molecular weight sugars, such as glucose, fructose, mannose, maltose, sucrose, and trehalose, which were much less abundant in samples collected from plants grown on AL as compared with DS or LL soil (Table 2). This is also reflected in the cumulative quantification of all sugars based on peak areas (Table 4), where the detected amounts increased in the order AL < DS < LL. The same trend was detectable for the cumulative quantification of amino acids but not for organic acids (Table 4).

**Table 4:** **Amounts of sugars, amino acids, and aliphatic organic acids in root exudate samples collected from 2-cm sub-apical root zones of lettuce, *Lactuca sativa* L. cv. Tizian (BBCH 19) grown on three different soils (calculations based on cumulative peak area after subtraction of background levels in bulk soil samples)**.

**Class of compounds**	**Alluvial loam**	**Diluvial sand**	**Loess loam**
Sugars	2.95	103.60	427.60
Amino acids	1.39	6.05	22.66
Organic acids	2.83	0.05	4.44

Urea was detected in similar amounts in soil solutions collected from the rhizosphere and from the bulk soil, while phosphate was present exclusively in rhizosphere soil solutions (data not shown).

The plants did not show any macronutrient disorders, since all soils received a standard N, P, K, Mg fertilization prior to the onset of the experiment. Also micronutrient concentrations (Table 5) were above the critical levels reported for lettuce by Bergmann (1988). No toxicities, apparent symptoms of diseases or pH extremes were observed and the soil pH ranged between 6.6 and 7.6. The soil moisture level was adjusted gravimetrically in regular intervals between 18–20% (w/w), equivalent to 70% of the water holding capacity, which is considered as optimal for plant growth.

**Table 5:** **Micronutrient concentrations in leaf dry matter (DM) of young lettuce *Lactuca sativa* L. cv Tizian (BBCH 19), grown on three different soils**.

**Soil type**	**Fe [mg kg^−1^DM]**	**Mn [mg kg^−1^DM]**	**Zn [mg kg^−1^DM]**
Loess loam (LL)	180.2 (8.6)	97.1 (4.2)	47.8 (0.6)
Diluvial sand (DS)	308.5 (8.5)	81.9 (16.2)	42.8 (2.1)
Alluvial loam (AL)	74.3 (5.3)	35.0 (3.2)	34.5 (4.2)

Means and SEs (in brackets) of four independent replicates.

## Discussion

Higher plants exhibit an enormous adaptive plasticity of root growth and morphology in response to external biotic and abiotic stress factors. Root growth stimulation particularly of fine root structures is frequently induced by moderate limitations of P, N, Fe, and water but lateral root development and root hair proliferation can be also stimulated by localized patches of high P, NO^−^_3_, and NH^+^_4_ supply. By contrast, root growth inhibition is a typical response to extreme limitations of water and nutrients, toxicities, and increased mechanical impedance induced by extreme drought or soil compaction (Neumann and Römheld, 2002).

However, in the present study, high variability observed in root growth of lettuce on three different field soils cannot be attributed to macronutrient disorders since all soils received a full N, P, K, and Mg fertilization prior to the onset of the experiment. Mineral nutrient analysis revealed soil-specific differences in the plant nutritional status but no apparent nutrient deficiencies or toxicities. Cropping history of the soils during the last 10 years was identical and no symptoms of plant diseases were visible. The largest differences in root length development were detected between the two similarly structured loamy soils (AL and LL), suggesting that also potential effects of soil structure on root growth are not the major cause for the observed variability in root growth. Moreover, the influence of soil structure was further minimized by homogenous sieving of all soils with 2 mm mesh size prior to the experiment. Therefore, the huge differences observed in root growth and morphology on the three investigated soils were unexpected.

Interestingly, Schreiter et al. (this issue) reported the highest number of bacterial rhizosphere responders in the rhizophere of lettuce grown on AL followed by DS and finally LL soils, which exactly reflects the order of root length development in the different soils (Table 2). Root growth stimulation of host plants by bacterial production of phytohormones (e.g. auxins) is a well-documented mechanism for plant-growth promotion by rhizosphere bacteria (Berg, 2009) and various bacterial genera, such as *Rhizobium* and *Pseudomonas* with known potential for root growth stimulation by auxin production (Biswas et al., 2000; Iqbal and Hasnain, 2013) were among the rhizosphere responders detected with the highest abundance in AL soil (Schreiter et al., this issue). This raises the question whether the observed differences in root growth and fine root production of lettuce on the different soils may be the result of differences in the abundance of root growth-promoting rhizobacteria. More fine-root development, resulting in a larger root surface area would in turn also provide increased space for root colonisation by rhizosphere responders.

### Root exudates

Apart from variability in root development, also huge quantitative differences particularly for sugars and amino acids were detected in the root exudate samples collected from lettuce plants, grown on the three different soils (Table 5). Comparable variations in root exudation over 1–3 orders of magnitude are characteristic in some plant species for the adaptive release of specific compounds, such as carboxylates or phytosiderophores involved in the mobilization of sparingly soluble mineral nutrients but also for detoxification of toxic elements or in response to membrane damage due to severe nutrient limitations or drought stress (Neumann and Römheld, 2007). However, the presence of nutrient deficiencies/toxicities or of other stress factors, limiting water and nutrient uptake was not indicated by soil and plant analysis (Tables 1, 5) and cannot explain the huge quantitative differences in root exudation. The fact that the samples collected from different soils exhibit mainly quantitative differences, while the qualitative composition was very similar (Table 3) suggests, that the detected compounds represent mainly root exudates and not microbial metabolites. In the latter case, a much higher qualitative diversity would have been expected due to the large variation of microbial populations in the different soils (Schreiter et al., this issue) which would consequently be reflected in a high diversity of microbial metabolites released into the rhizosphere.

However, root exudate sampling was performed over a time period of 4 h to increase the chance of collecting also less polar aromatic compounds and other secondary metabolites, usually less abundant in root exudates (Neumann, 2006b). On the other hand, longer sampling periods are associated with a risk of losses of readily soluble exudate compounds, such as sugars, amino acids, and organic acids, which are easily used as carbon and N sources by rhizosphere microorganisms (Neumann, 2006b), resulting in half life times of only several hours in rhizosphere soil solutions (Jones et al., 1996). Drastically reduced levels of sugars and amino acids in exudate samples collected from AL as compared with DS and LL soils, may therefore reflect a particularly intense microbial consumption of these compounds as a consequence of a higher abundance of bacterial rhizosphere responders detected by Schreiter et al. (this issue) in the rhizosphere of AL-grown plants. Differences in the root exudate profiles collected from lettuce plants grown on the different soils were further confirmed by PCA (Figure 2).

Another interesting finding is the detection of benzoic and lauric acids in the root exudate samples. Both compounds have been previously reported as constituents of root exudates of lettuce in hydroponic culture with (auto-)allelopathic (Lee et al., 2006) and antifungal properties (Walters et al., 2003; Yoon et al., 2012). Moreover, the rhizosphere responders (*Sphingomonas*, *Pseudomonas*, and *Variovora)* detected by Schreiter et al. (this issue) in the rhizosphere of lettuce grown on the same soils exhibit a high potential for degradation of aromatic hydrocarbons, such as benzoic acid and De la Fuente et al. (2009) reported the ability of various *Pseudomonas* strains to utilize trehalose and benzoic acids as sole carbon sources, both detected in the root exudates of lettuce in this study.

Urea detected at similar levels, both in bulk soil and root exudate samples (data not shown) cannot be attributed to N fertilization since nitrate was applied as a mineral N source. The presence of urea in all analyzed samples may reflect a background level of microbial urea production, which has been previously confirmed by isotope studies also for other agricultural soils (Nielsen et al., 1998). By contrast, phosphate, detected only in root exudate samples may indicate preferential mobilization of weakly adsorbed phosphate in the rhizosphere, mediated by plant roots or rhizosphere microorganisms (Neumann and Römheld, 2002).

In summary, the present study suggests that even under controlled conditions on well-fertilized soils, excluding the influence of cropping history and stress factors with impact on root growth and activity, mutual interactions between plant roots, and soil-specific microbiomes seem to be important determinants for shaping root architecture, root exudation and thereby the establishment of rhizosphere-microbial communities.

### Conflict of interest statement

The authors declare that the research was conducted in the absence of any commercial or financial relationships that could be construed as a potential conflict of interest.
